# Impacts of seasonal flooding on geographical access to maternal healthcare in the Barotse Floodplain, Zambia

**DOI:** 10.1186/s12942-023-00338-3

**Published:** 2023-07-31

**Authors:** Elizabeth Jade Mroz, Thomas Willis, Chris Thomas, Craig Janes, Douglas Singini, Mwimanenwa Njungu, Mark Smith

**Affiliations:** 1 School of Geography and water@Leeds, University of Leeds, Leeds, LS2 9JT UK; 2 Lincoln Centre for Water & Planetary Health, University of Lincoln, Lincoln, LN6 7DW UK; 3 School of Public Health Sciences, University of Waterloo, Waterloo, ON N2L 3G1 Canada

**Keywords:** Geographical information systems, Network analysis, Cost distance analysis, Maternal health services, Access to healthcare, Seasonal floods, Health geography

## Abstract

**Background:**

Seasonal floods pose a commonly-recognised barrier to women’s access to maternal services, resulting in increased morbidity and mortality. Despite their importance, previous GIS models of healthcare access have not adequately accounted for floods. This study developed new methodologies for incorporating flood depths, velocities, and extents produced with a flood model into network- and raster-based health access models. The methodologies were applied to the Barotse Floodplain to assess flood impact on women’s walking access to maternal services and vehicular emergency referrals for a monthly basis between October 2017 and October 2018.

**Methods:**

Information on health facilities were acquired from the Ministry of Health. Population density data on women of reproductive age were obtained from the High Resolution Settlement Layer. Roads were a fusion of OpenStreetMap and data manually delineated from satellite imagery. Monthly information on floodwater depth and velocity were obtained from a flood model for 13-months. Referral driving times between delivery sites and EmOC were calculated with network analysis. Walking times to the nearest maternal services were calculated using a cost-distance algorithm.

**Results:**

The changing distribution of floodwaters impacted the ability of women to reach maternal services. At the peak of the dry season (October 2017), 55%, 19%, and 24% of women had walking access within 2-hrs to their nearest delivery site, EmOC location, and maternity waiting shelter (MWS) respectively. By the flood peak, this dropped to 29%, 14%, and 16%. Complete inaccessibility became stark with 65%, 76%, and 74% unable to access any delivery site, EmOC, and MWS respectively. The percentage of women that could be referred by vehicle to EmOC from a delivery site within an hour also declined from 65% in October 2017 to 23% in March 2018.

**Conclusions:**

Flooding greatly impacted health access, with impacts varying monthly as the floodwave progressed. Additional validation and application to other regions is still needed, however our first results suggest the use of a hydrodynamic model permits a more detailed representation of floodwater impact and there is great potential for generating predictive models which will be necessary to consider climate change impacts on future health access.

**Supplementary Information:**

The online version contains supplementary material available at 10.1186/s12942-023-00338-3.

## Background

Geographical barriers to healthcare facilities are linked to poor uptake of maternal health services, and worsened maternal and neonatal outcomes [1–5]. Geographical health access models can be created using GIS (geographical information systems) software [6–15]. These models can account for the impact of geographical barriers on health access when calculating population travel times and distances to health services. This approach is commonplace in maternal health research as geographic health access models are able to identify underserved populations, provide evidence to support interventions on improving accessibility, and identify the most influential geographical barriers to access [16–19]. For example, Ruktanonchai et al. [1] used cost-distance analysis as part of their study investigating maternal healthcare utilisation, and found that increased geographical inaccessibility had the strongest association with reducing maternal care usage in five East African countries.

Many geographical barriers are static, permanent features on the landscape such as rivers, mountains, and roads. However, flooding is an important temporary geographic barrier which can impact access [11, 20–22]. In low-income countries with distinctive seasonal climates, floods are a regular impactful barrier to accessing health facilities [11, 16, 17, 23–26]. Many studies have recognised floods as an important geographical barrier [11, 21, 24–26] as they can persist for long periods of time, restricting health access and isolating entire communities. They also impact the supply-side of health access by causing medical supply chain shortages and hindering referrals [24, 27]. As a result, in the wet season, morbidity and mortality can be increased compared to the dry season [18, 28–32]. Despite the detrimental impacts of floods on health systems, few studies have considered or modelled the impacts of floods on health access (see [11, 17, 33] for examples). Failure to account for floods leads to models that over-estimate accessibility.

There is a dearth of research on how floods directly impact access through space and time. Blanford et al. [17] presented contrasting maps of dry and wet season access which emphasised significant drops in accessibility due to seasonal impacts, but the maps were otherwise static representations which limited understanding of how access changes between the peak dry and peak wet season as well as the timings of the transitions throughout intermediate flood stages. Makanga et al. [11] pioneered the first spatio-temporal framework for assessing access changes under seasonal conditions using daily records of precipitation and satellite-derived flood extents. Whilst this approach substantially advanced access models, there persists a limited understanding of the relationship between floodwater variables (depth and velocity in addition to extent) and access. Extents can only be treated as impassable barriers in the absence of additional data on depth and velocity that can be utilised to characterise flood hazard. However, not all floodwaters will be equally inaccessible; some floodwaters may be a few centimetres deep and thus navigable at reduced speeds, whilst others may be metres deep. Consequently, including these additional hydrodynamic variables would improve the real-world representativeness of floodwaters in access models. This is advantageous as it better constrains model estimations on the duration of accessibility impact, which populations are isolated, planning medical supplies, and assessing referral capabilities.

This study aims to improve the representation of floodwaters in health access models through a new methodology. For the first time, we couple vector- and raster-GIS-based access models with a hydrodynamic flood model to create novel geospatial frameworks that utilise the hydrodynamic variables of flood depth and velocity. The new framework is applied to the Barotse Floodplain, Zambia, which presents an appropriate case study to investigate two critical scenarios of access to maternal services, for which access models are routinely applied:

A) The ability of women of reproductive age to walk to their nearest healthcare facility (HF) offering maternal services (raster-based model).
B) The ability of delivery sites (facility providing a trained birth attendant) to make pregnancy-related emergency referrals to the nearest emergency obstetric care location (EmOC) by vehicular road transportation (vector-based model).

The following objectives were implemented to assess these scenarios:

**Objective 1:** To calculate walking travel times for women to their nearest maternal services, and to calculate driving travel times for emergency referrals between delivery sites and EmOC sites.

**Objective 2:** To calculate the number and percentage of women that have timely walking access within 2-hours to maternal services, and that can be referred within a timely 1-hour from a delivery site to an EmOC site.

**Objective 3:** To assess the impact of floods on increasing walking and driving travel times, and how that affects the number and percentage of women considered to have timely access to maternal services.

**Objective 4:** To quantify the number and percentage of women who become inaccessible due to floodwater impact as it varies in each month.

**Objective 5:** To evaluate the interaction between the ability of women to walk to the nearest delivery site and the referral system.

## Methods

### Study area

**Fig. 1:**
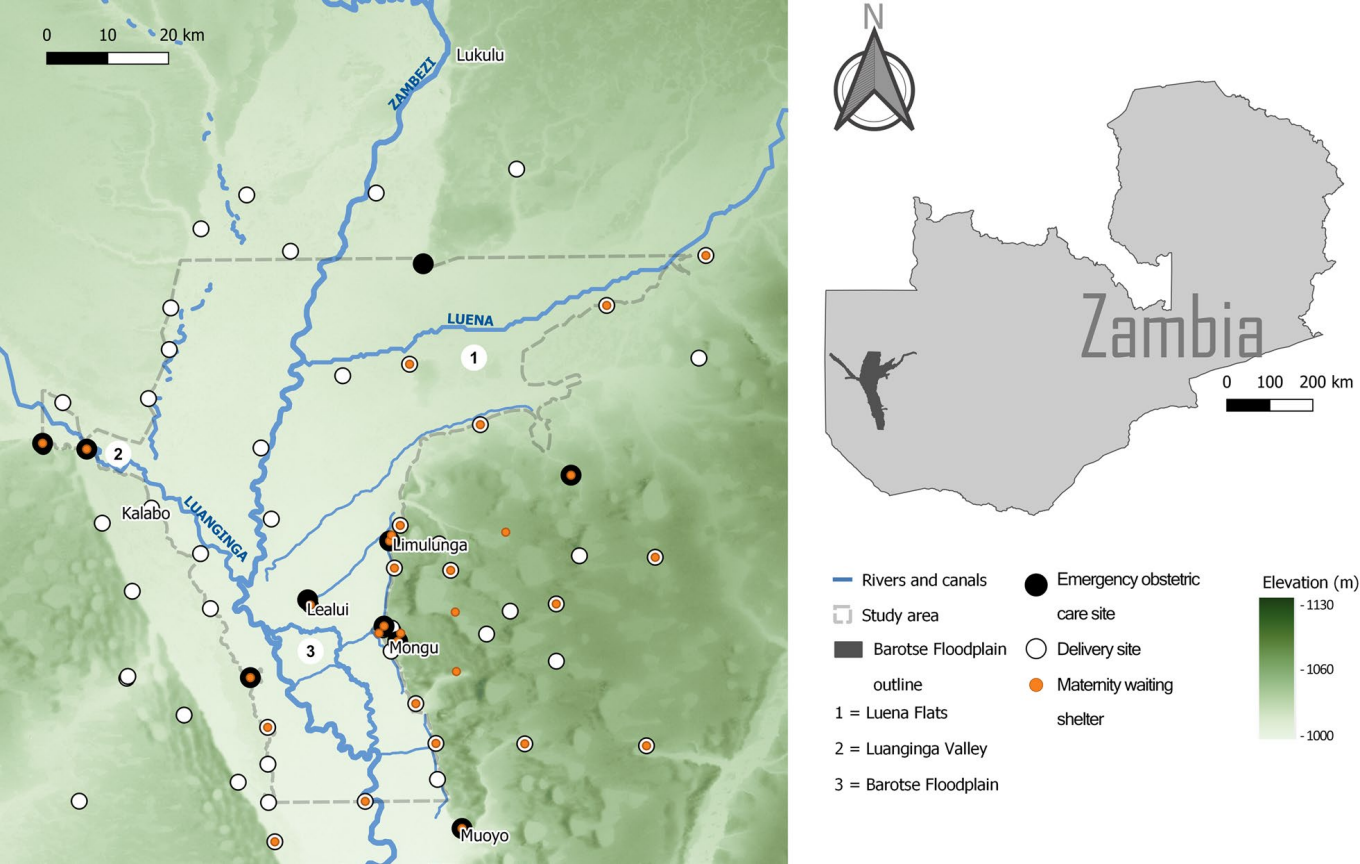
Map of the Barotse Floodplain and its major waterbodies, and the floodplain’s location within Zambia

The Barotse Floodplain is a large African floodplain located in the Upper Zambezi valley, Zambia (Fig. 1). Due to the movements of the Inter-Tropical Convergence Zone (ITCZ), the Zambezi has a strongly seasonal discharge which causes the floodplain to experience annual floodwaves. The amount of precipitation that falls in the upstream catchment is significant in determining the magnitude of each floodwave, and the timing of upstream precipitation controls the timing of flooding as there is a delay of approximately two months between peak rainfall and peak inundation ([34]; Additional file 1: Figure S1). Extensive, slow-moving floodwaters typically persist between February and June due to the floodplain being extremely flat and attenuating flow [35]. The peak of the floods occurs in late March or early April where the total inundated area can be as great as 10,750 km^2^ [36].

The floodplain is inhabited by the semi-nomadic Lozi people, whose population within the study area (Fig. 1) is estimated using the High Resolution Settlement Layer (HRSL) to be ~ 44,600 as of 2021 [37]. With the exception of the Barotse Floodplain Causeway, roads on the floodplain are unpaved and only navigable by four-wheel drive vehicles (4WD) or motorcycles [38]. The Lozi predominantly move on foot, by oxen-cart, and dugout canoes on open waterbodies [39–41]. Oxen-cart and dugout canoes are influential local modes of transport but were not incorporated in this study due to a lack of data to characterise their usage and due to a lack of suitable methods for incorporating boats into access models [42–45].

Access models require data to understand the combination of transport modes, travel speeds, and also other influential variables such as financial cost and waiting times [15, 44, 46–49]. It is common for access models to represent transport as individualised rather than multimodal in the absence of such data to prevent incurring significant assumptions and uncertainties [46, 50]. The walking model is therefore a conservative measure of access as individuals potentially have access to faster modes of transports, which the walking model doesn’t account for and consequently assumes longer travel times. This is preferable to a model that may include multiple methods of transportation but without substantial data to support its parameterisation, consequently introducing unknown variability that may overestimate accessibility. Oxen-carts have similar travel speeds to walking [51–53] so their usage was assumed to be represented adequately in the walking model.

Maternal mortality is a key health concern on the floodplain, with previous studies indicating that Western Province has the highest maternal mortality ratio compared to any other province in Zambia [54, 55]. Efforts are ongoing to increase the number of births at health facilities as a mitigation strategy. Whilst many other socioeconomic and sociodemographic factors also contribute to poor maternal services uptake and consequently poor maternal outcomes [56], geographical access during floods are the primary focus. The annual floods complicate mitigation initiatives as they obstruct women accessing care, hinder emergency referrals, and cause difficulties in ensuring facilities remain operational [38].

### Locations of maternal services and women of reproductive age

A geolocated dataset of public health facilities on and surrounding the floodplain (*n* = 75) was acquired from Zambia’s Ministry of Health Master Facility List (MFL) [57]. The MFL provides no information on services provided at facilities, so the 2012 List of Health Facilities in Zambia [57] was used to supplement information on facility type and maternal services (Table 1); this document contained the most up-to-date information available. For facilities constructed since 2012 (*n* = 25), their services were assumed by assigning the most common services typically offered by health facilities of the same type; the majority were health posts (the lowest level of healthcare catering to distant communities). EmOC can be classified as either basic or comprehensive, depending on the presence of specific signal functions (major interventions for reducing maternal and neonatal mortalities) [58]. The 2012 facility list makes no reference to signal functions, so EmOC is assumed to be at least basic for all facilities and to meet UN standards. Data on women of reproductive age as of August 2021 were obtained from the HRSL dataset [37]. HRSL provides gridded population density data at a spatial resolution of 30 m. The HRSL data were estimated using a binary top-down modelling approach where census data were proportionally attributed to buildings identified through convolutional neural networks operating on satellite imagery with a spatial resolution of 50 cm [37]. For the purpose of this study, these were converted into geolocated points representing the number of women (*n* = 8,130) living within each 30 m grid cell of the study area.

**Table 1:** Description of key maternal services offered by healthcare facilities in Zambia [57], and the number of facilities providing each service within a 15 km vicinity of the floodplain study area

Maternal service	Description	Number of facilities
Delivery site	A healthcare facility able to provide a trained/skilled birth attendant to assist during labour.	65 (*35 urban, 30 rural)*
Emergency obstetric care (EmOC)	The provision of medical care able to assist with life-threatening obstetric complications such as haemorrhage, a ruptured uterus, and eclampsia.	10 *Situated in urban areas*
Maternity waiting shelter (MWS)	Accommodations provided for pregnant women so that they can remain close to a delivery site in anticipation of labour.	33 (*12 urban, 21 rural*)

No complete road network dataset was available for the Barotse Floodplain, so floodplain roads were manually delineated by visual inspection of very high resolution (< 5 m) composite satellite imagery sourced in the dry season from Google Earth and Microsoft Bing Maps. Delineation was conducted between October 2019 and October 2020, with additional checks of network completeness conducted in April, June, and July 2021 before the dataset was used. Roads were classified as either primary, secondary, track, elevated highway, or water crossing. Road type was unpaved, with the exception of the Barotse Floodplain Causeway (an elevated highway running across the floodplain). Beyond the floodplain, OpenStreetMap (OSM) [59] data were sourced in June 2021 and recoded to match the same classification as the floodplain roads (Additional file 1: Table S1). Gaps in OSM coverage were resolved through manual delineation. GPS tracks of recorded journeys across the floodplain were used to validate the accuracy of the delineation procedure. Additional ground-truthing was provided by local health officers who provided guidance on the local environment.

### Permanent waterbodies and floodwaters

A shapefile of the positions and average widths of rivers and canals on the floodplain were provided by Willis et al. [60]. These waterbodies are permanent and hence were modelled as static barriers to access. To convert the waterbodies shapefile to raster, the waterbodies were first buffered according to their average width and then rasterised. Non-static, temporary floodwaters were obtained through flood modelling. The LISFLOOD-FP hydrodynamic inundation model [61] was run for the Barotse Floodplain between October 2017 and October 2018 to produce monthly rasters containing information on floodwater depth (m), velocity (m s^− 1^), and extent (m^2^). The 2018 hydrological year was selected for modelling as the floodwave was one of the largest experienced in recent years yet remained within the expected interannual flood magnitude, and the temporal aspect of the floodwave was characteristic of a typical year [62].

The flood model set up is described by Willis et al. [60] and was configured to represent the hydrological processes operating on the floodplain, assuming flows are mostly subcritical and low velocity. Terrain data for the flood model were obtained from a high-resolution TanDEM-X1 Digital Elevation Model (DEM) from 2016, which has a vertical accuracy of ~ 2 m on the floodplain and a native spatial resolution of 12 m [63] but was resampled to 100 m for the flood model due to computational costs. The model was calibrated and validated using both gauge data provided by the Water Resources Management Agency (WARMA) and remotely-sensed flood extents derived from Landsat (30 m spatial resolution). Willis et al. [60] demonstrated that the model reproduced the characteristics of the 2018 floodwave, with its best performance occurring at the flood peak (goodness of fit, F^2^ = 0.62). Lower performance metrics were returned during the intermediate flood stages (F^2^ = 0.10); however, these values likely reflect issues with using satellite-derived flood extents for validation. Optical sensors such as Landsat are unable to recognise vegetated waters, so areas denoted as “false positive inundation” during validation are likely truly inundated but not detectable by Landsat [64].

### Modelling access to care: walking

**Fig. 2:**
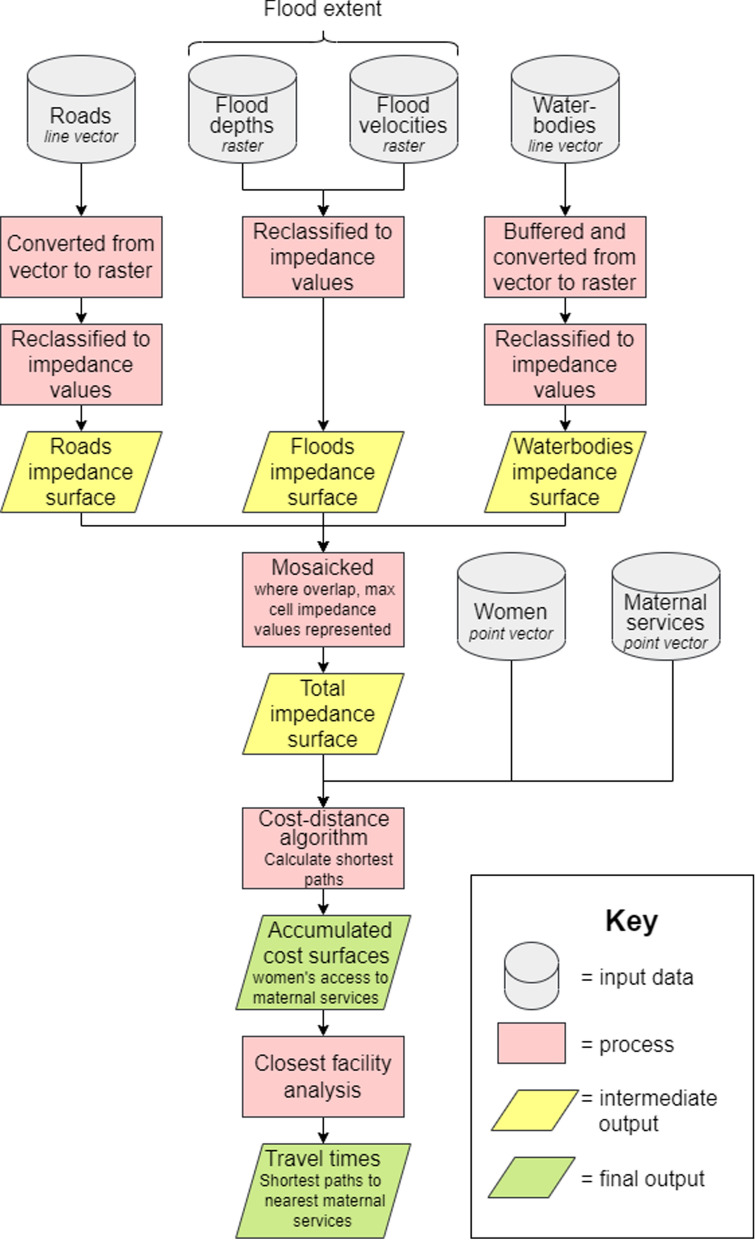
Simplified flow diagram of the raster-based model framework. Maternal services are either delivery sites, emergency obstetric care (EmOC), or maternity waiting shelters (MWS)

A semi-automated raster-based model was created that estimated walking travel times for women to their nearest delivery site, EmOC location, and maternity waiting shelter respectively (Fig. 2). Travel times were computed from each populated point (placed at the centroid of the 30 m cells containing a population in the gridded HRSL dataset) rather than aggregating the HRSL population data into a coarser geographic unit. Women were assumed to walk to the nearest healthcare facility offering a maternal service rather than bypass in favour of facilities further away.

Impedance surfaces were first created in an automated workflow within FME Workbench 2020.2 [65, 66]. Impedance (also known as friction) surfaces are a grid of cells representing the ease of movement across a geographical landscape; each cell contains a value which denotes how difficult it is to move through [13, 67]. In this study, impedance surfaces with a 10 m spatial resolution were output at a monthly timestep by mosaicking together data on roads, permanent waterbodies, and the corresponding month’s floodwater rasters representing depth, velocity, and extent. The flood rasters were used to determine whether flood hazard was great enough to render an area inaccessible. In the walking scenario for pregnant woman, areas were modelled as impassable if flood depths exceeded 1 cm. Where data overlapped when creating the impedance surface, the maximum impedance value was represented in the final surface. Slope and land use data were not considered due to the floodplain being extremely flat with a near-homogenous land use. A walking speed of 5 km hr^− 1^ is typically used to represent an average walking speed in the absence of topographical impediments [2, 12, 68–71]. In this study walking speeds were parametrised to reflect a decreased mobility of pregnant women: 4 km hr^− 1^ for on-road travel, 3 km hr^− 1^ for off-road travel, and 0 km hr^− 1^ for areas that were flooded as any cells that contained floodwaters exceeding 1 cm depth were considered to be completely impassable. Flooded cells were completely removed from the impedance surfaces so that they could not be routed through.

The monthly impedance surfaces were then transferred to QGis Desktop 3.16.6 with GRASS 7.8.5. The cost-distance algorithm *r.cost* [72] was used to compute monthly minimum isotropic accumulated cost surfaces of access [12, 67, 73]. These surfaces were computed from the population points to delivery sites, EmOC, and MWS respectively. The *r.cost* algorithm was selected due to its superior accuracy over other cost-distance algorithms resulting from movement calculations occurring over 16-cells rather than 8-cells [74–77]. The cost surfaces were discretised into travel time thresholds with 2-hours identified as the critical threshold representing timely access to a maternal service [46, 78–80]. A closest facility analysis was then performed by extracting the shortest path travel times for each populated point and identifying health facility service areas.

### Modelling access to care: driving

**Fig. 3:**
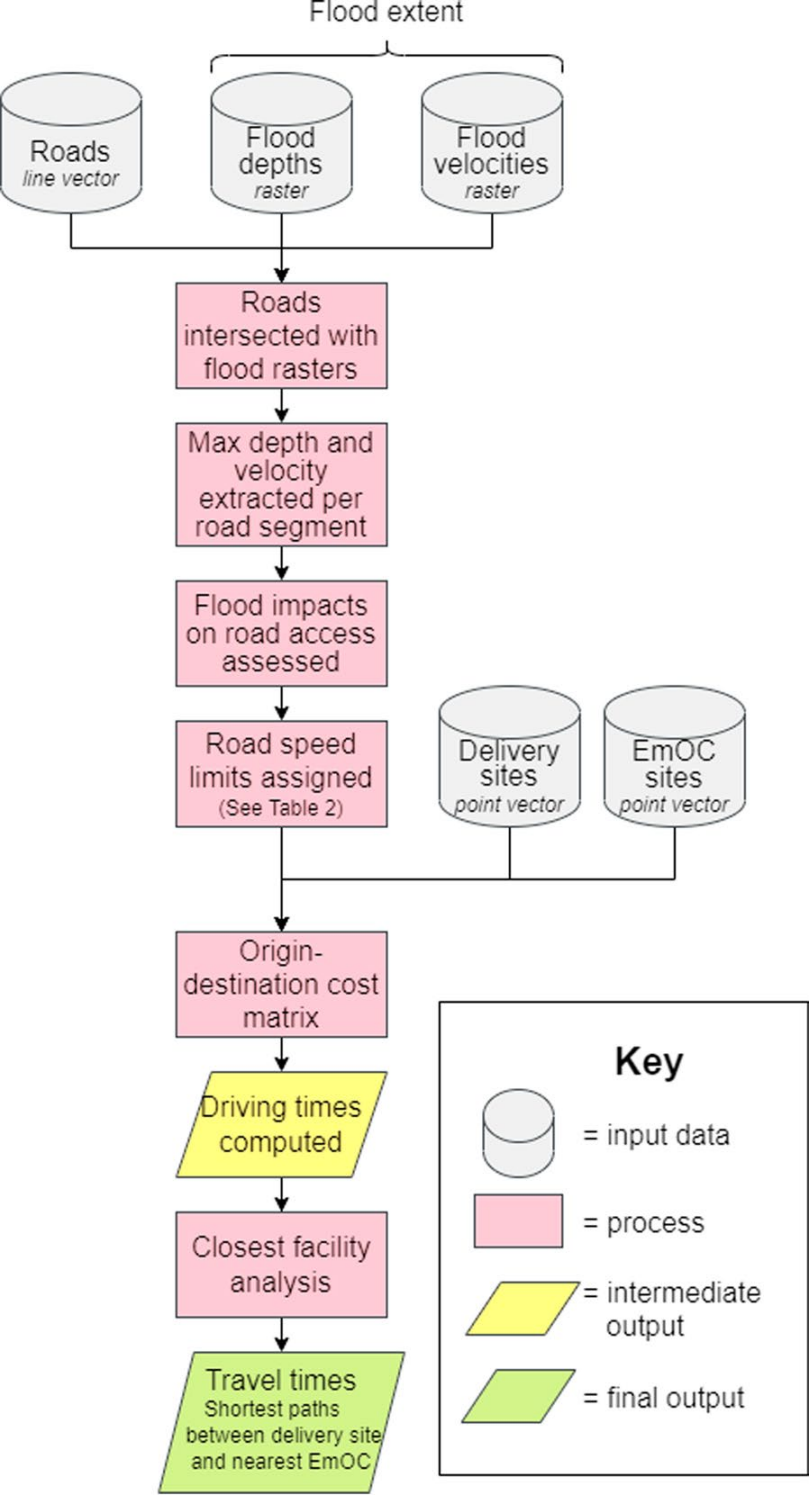
Simplified flow diagram of the vector-based model framework

An automated network-based model was created in FME Workbench 2020.2 to calculate emergency referral travel times between delivery sites and emergency obstetric care locations (Fig. 3). The referral chain modelled assumes that women walk to their nearest delivery site, experience obstetric complications during labour, and are then immediately referred to the nearest EmOC location by an emergency 4WD vehicle. Hence, emergency referral times refer explicitly to the time taken to drive between delivery sites and EmOC locations, and there is no consideration of referrals for women who experience obstetric complications at home and may bypass facilities to immediately access EmOC.

Maternal referral systems, including Zambia’s, are typically hierarchical and designed for healthcare practitioners to make referrals [81–83] with practices in place that can result in discouraging self-referrals [84]. Hence, referral models commonly are set-up to observe only referral pairs of sending and receiving facilities, with no consideration of women bypassing facilities to self-refer [85, 86].

 To calculate travel times, speed limits were first assigned for each road segment in the road network dataset (Table 2 ; [87]) whilst speed limits do not apply to emergency vehicles, the values represent maximum possible speeds along the different road conditions. To account for the impacts of floodwater variables on road accessibility, the road network dataset was intersected at a monthly time interval with the corresponding floodwater depth and velocity rasters (Fig. 3*)*.

The updated road network dataset for each month thus designated speed limits by the monthly flooded status of each road. If no floodwaters intersected a road segment, it was assumed to be dry and unflooded, so a speed limit was assigned based upon Zambia’s speed limit regulations [87]. If floodwaters intersected a road segment, a series of conditional statements were used to determine the impact to accessibility (either a speed limit reduction or complete impassability) (Table 2).

The conditional statements use depth and velocity thresholds collated from literature on vehicular instability to determine the hazard that floods pose to a 4WD vehicle [88–92]. Floodwater depth and velocity are the two primary hydrodynamic variables that determine flood hazard to a vehicle, so assessment of both is critical. For example, floodwaters with shallow water depths and high velocities are equally as hazardous as deep floodwaters with low velocities. The thresholds provided by the Australian Rainfall and Runoff Project [93] are considered the most suitable and best reference for stability criteria [94] as nearly all experiments show vehicular instability to occur at depths and velocities greater than the criteria (Additional file 1: Table S2). In this study, we followed the guidance of the Australian Rainfall and Runoff Project in setting a maximum flood depth stability criteria of 0.5 m, but imposed a more conservative maximum flood velocity stability criteria of 1.0 m s ^− 1^. The resulting statements assume that: drivers have omniscient knowledge of floodwater properties and the hazard they pose to their vehicle; drivers will drive through floodwaters where theoretically safe to do so; depth and velocity are the only variables important to vehicular stability; all emergency referrals use a 4WD vehicle; all road conditions are equal; and drivers always drive safely and at appropriate speed.

Roads are considered dry and accessible at usual speed limits when flood depth is 0 m and flood velocity is 0 m s^− 1^. Roads are considered wet when flood depth is greater than 0 m but less than 0.01 m, and speed limits are reduced by a third. Roads are denoted as flooded when flood depth is greater than 0.01 m but less than 0.1 m, and deeply flooded when flood depth is greater than 0.1 m but less than 0.5 m, with speed limits drastically reduced. Roads become impassable when either the absolute maximum flood depth threshold of 0.5 m or maximum flood velocity threshold of 1.0 m s^− 1^ are exceeded.

**Table 2:** Speed limits assigned to roads in the network-based model. D refers to floodwater depth, whilst V refers to floodwater velocity

Road type	Travel speed (km hr^− 1^) in flood conditions:				
	Dry and unflooded	Wet	Flooded	Deeply flooded	Impassably flooded
	*D = 0 m* *V = 0 m s* ^*− 1*^	*D = < 0.01 m*	*0.1 m > D > 0.01 m*	*0.5 m > D > 0.1 m*	*D = > 0.5 m OR* *V = > 1.0 m s* ^*− 1*^
Primary	80	26.7	8	5	0
Secondary	50	16.7	8	5	0
Track	30	10	8	5	0
Elevated highway	120	n/a	n/a	n/a	n/a
Watercrossing	15	5	8	5	0

For each month, an origin-destination cost matrix was then computed between every delivery site and every EmOC location, using Dijkstra’s algorithm to calculate travel times [95]. A closest facility analysis was performed to identify the shortest travel time to EmOC for each delivery site, and the corresponding path and identity of the closest facility. A one hour travel time threshold was used as an indicator of timely access to life-saving emergency obstetric treatment; this “golden hour” is commonly used as an indicator of timely access to EmOC due to drastically reduced morbidity and mortality outcomes if EmOC is administered within an hour [96–98]. Health facilities were considered inaccessible and unable to make or receive referrals if they were disconnected from the road network, whether by floodwater impact or simply due to a lack of existing road connection.

Identifying potential referrals between delivery sites and EmOC is complicated by the multiple impacts of floods on different parts of the referral system. Firstly, women must be able to walk to a delivery site, and the proportion of women with access alters due to floodwave passage. Secondly, the positioning of floodwaters alters which delivery sites are being accessed each month; this in turn changes the number of women served by facilities each month. Finally, floods directly impact the ability of delivery sites to make referrals to EmOC. Consequently, herein we accounted for these complications by explicitly considering only referral coverage and referral times in each month for the delivery sites that the walking model showed were being accessed by women. This criterion weighted the referral results based upon the impact to the floodplain population.

## Results

### Characterisation of the 2018 floodwave

The results showed that both walking times and referrals were impacted monthly between October 2017 and October 2018 due to the changing depths, velocities, and extent associated with the passage of the annual floodwave (Additional file 1: Table S3). Consequently, characterising the floodwave dynamics was important to the interpretation of the access results.

**Fig. 4:**
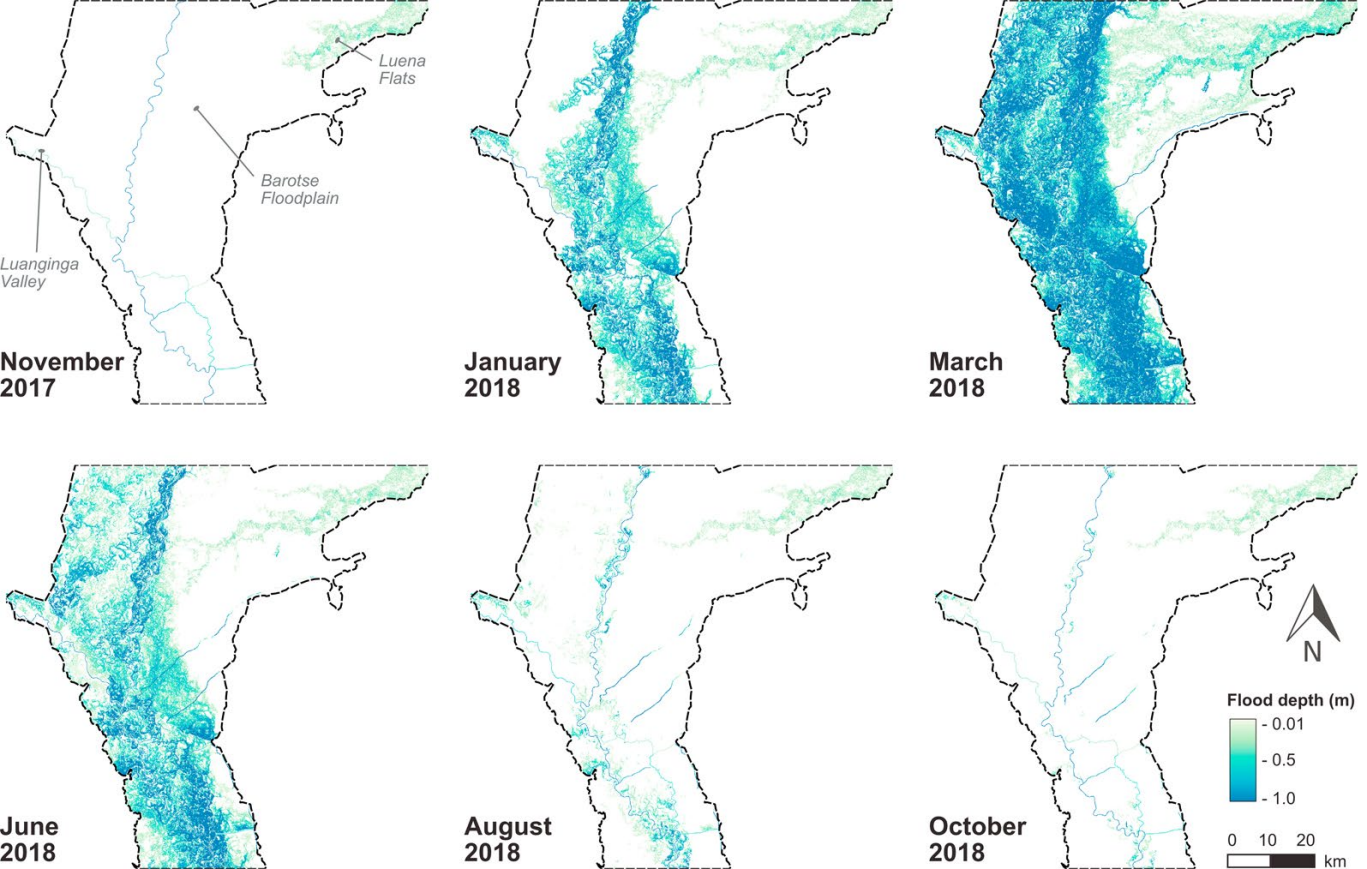
Modelled floodwater extent and depth in six selected months of the total 13 months

Floodwaters first appear in the Luena Flats in November 2017, with floodwater depths below 0.3 m and velocities below 0.2 m s^− 1^ (Fig. 4). By December 2017, floodwaters appear in the Luanginga Valley and Barotse Floodplain. All floodwaters increase in extent and depth from January 2018 until the flood peak in March and April 2018 when inundation extent is greatest and deep floodwaters (> 1 m) span to the escarpment of the Barotse Floodplain; floodwaters in the tributary valleys are extensive but shallower. From June 2018, flood drawdown occurs. By October 2018, floodwaters have mostly receded, but some shallow floodwaters (~ 0.2 m) persist in the Luanginga Valley and Luena Flats. Throughout the floodwave, velocities remain low, infrequently exceeding 0.5 m s^− 1^.

### Modelling access to care: walking

Timely access within 2 h was most optimal in the dry season month of October 2017. Of the 8,130 women of reproductive age living on the floodplain, 55% had timely access to their nearest delivery site, 19% had timely access to their nearest EmOC location, and 24% had timely access to the nearest MWS. Despite this being the peak month for access to maternal services, the average journey is long, and large disparity exists in travel times. The average walking times for women of reproductive age living on the floodplain were 109 min (1.8 h) to the nearest delivery site, 280 min (4.7 h) to the nearest EmOC location, and 251 min (4.2 h) to the nearest MWS. However, women located in the most remote areas of the floodplain must walk a maximum travel time of 420 min (7 h) to the nearest delivery site, and 845 min (14.1 h) to both the nearest EmOC location and MWS.

**Fig. 5:**
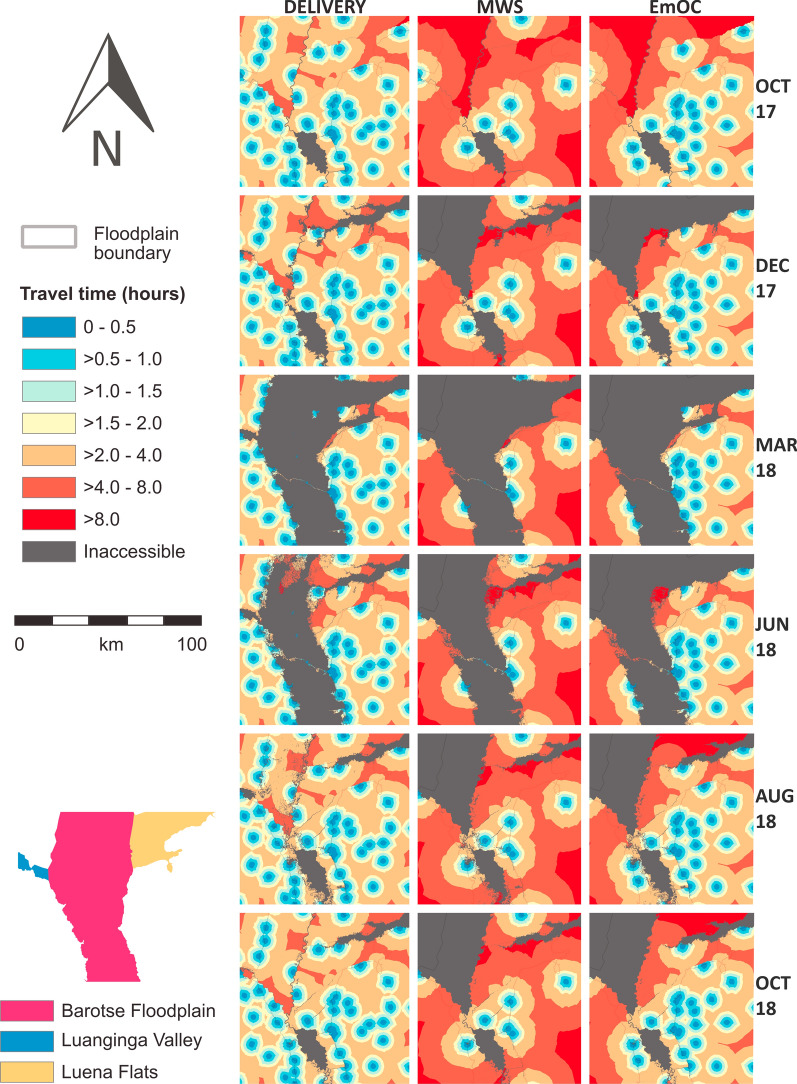
A comparison of accessibility to different maternal services in six selected months that the analysis was conducted between October 2017 and October 2018

**Fig. 6:**
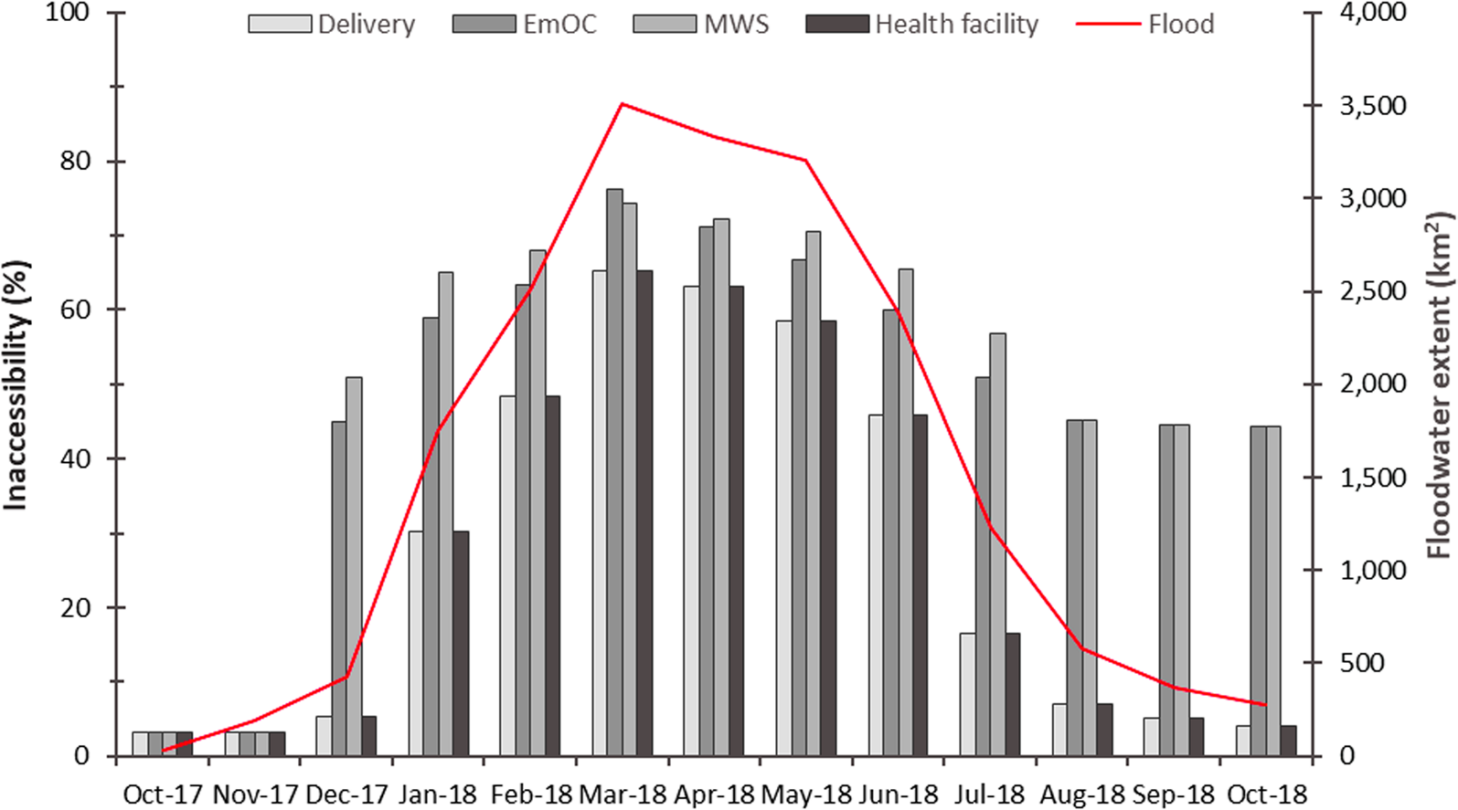
Percentage of women unable to walk to maternal services in each month the analysis was conducted between October 2017 and 2018. The red line indicates modelled floodwater extent as measured across the floodplain study area. Health facility refers to any facility that can be accessed irrespective of services provided

**Fig. 7:**
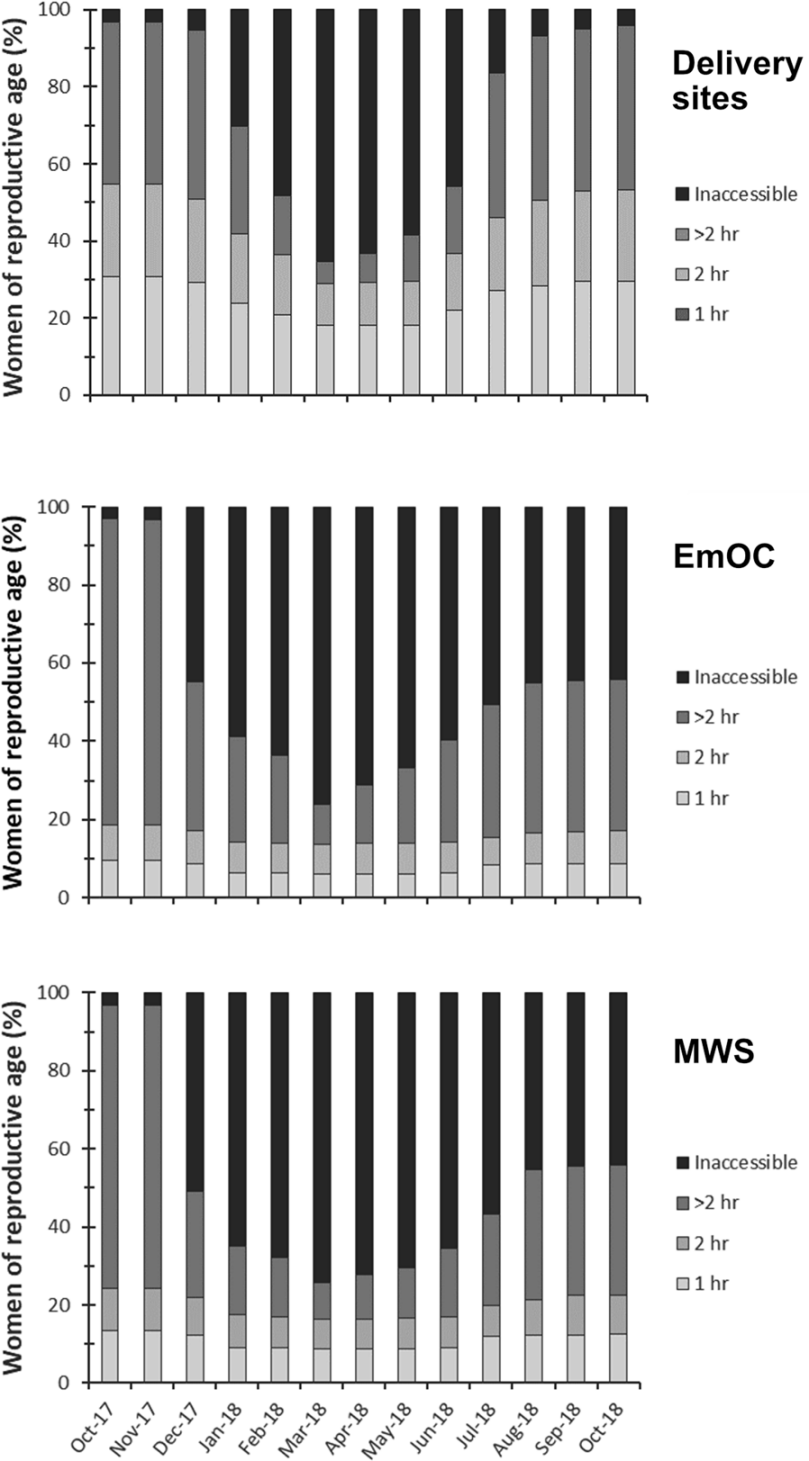
Percentage of women within each walking time threshold to different maternal services for each month the analysis was conducted between October 2017 and October 2018

The spatio-temporal distribution of floodwaters negatively impacted the ability of women to walk to their nearest maternal services through increases in travel times and increases in inaccessibility (Figs. 5, 6 and 7; Additional file 1: Table S4). By the peak of flooding in March 2018, the percentage of women of reproductive age with timely access within 2 h to their nearest delivery site, EmOC location, and MWS dropped to 29%, 14%, and 16% respectively. The majority of women had no access at all in March 2018, with 65% of women unable to access any delivery site, 76% of women unable to access any EmOC location, and 74% of women unable to access any MWS.

A higher walking time to the nearest facility in the dry season indicates an increased likelihood of experiencing inaccessibility at the peak of the floods. For women located more than two hours away from their nearest delivery site in October 2017, 89% became unable to access any delivery site by March 2018. In comparison, inaccessibility rose to a maximum of 33% in March 2018 for women who had access to the nearest delivery site within an hour in October 2017. Similar patterns exist for inaccessibility rates to EmOC and MWS. For women located more than 2 hours away from the nearest EmOC location in October 2017, 87% experience inaccessibility by March 2018, compared to 17% of women who have dry season access within an hour. For women located more than two hours away from the nearest MWS in October 2017, 89% experience inaccessibility by March 2018 compared to 14% of women who have dry season access within an hour.

There is temporal variation in both the onset and duration of flood impacts to the different maternal services. Rising floodwaters impact access to EmOC and MWS in December 2017, a month earlier than they impact access to delivery sites. In November 2017, 3% of the 8,130 women are considered unable to access any delivery site, EmOC or MWS. Inaccessibility to EmOC and MWS rises to 45% and 51% respectively in December 2017, whilst inaccessibility to delivery sites only rises to 5% across the same time period (Fig. 6*).* Similarly, elevated inaccessibility to EmOC and MWS persists from July 2018 onwards whilst accessibility to delivery sites is continually restored. Reductions in inaccessibility to EmOC and MWS completely stagnate from August 2018 until the final model timestep in October 2018 whilst inaccessibility to delivery sites has returned to its pre-flood values; this relates to the modelled continued presence of shallow floodwaters persisting in the Luanginga Valley, which impacts access to EmOC and MWS.

### Modelling access to care: driving

In the 2018 hydrological year, 40 healthcare facilities were the nearest delivery site in at least one month for women living on the floodplain. Of these, 7 also provided EmOC services and were assumed not to make road referrals to other EmOC locations. Of the 33 facilities that can only provide delivery services, four lack any direct road connection and were unable to make road referrals year-round.

October 2017 is the optimal month for emergency referrals with 65% of women modelled to have timely referrals within an hour from their nearest delivery site to EmOC. There are 27 health facilities that act as the nearest delivery site to women in October 2017, and all are able to make referrals. Of these, only 4 are unable to make referrals to the nearest EmOC location within an hour. The average referral time is 30 min, whilst the maximum referral time is 89 min.

**Fig. 8:**
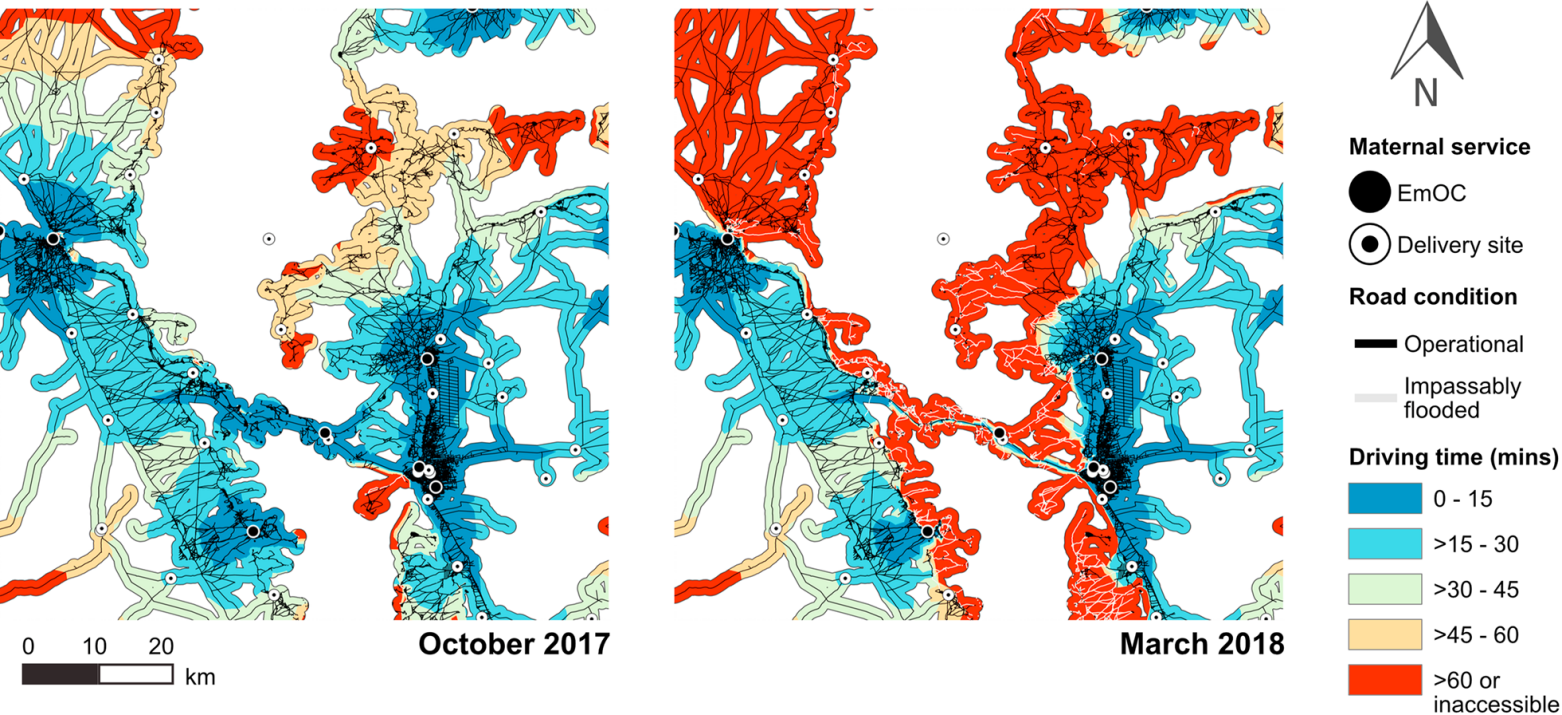
Vehicular referral times between delivery sites and EmOC locations compared. October 2017 is the optimal month for referrals, whilst March 2018 is the peak of flood impacts on referrals. Areas coloured orange denote routes outside of the “Golden Hour” whether due to high travel times or complete inaccessibility

**Fig. 9:**
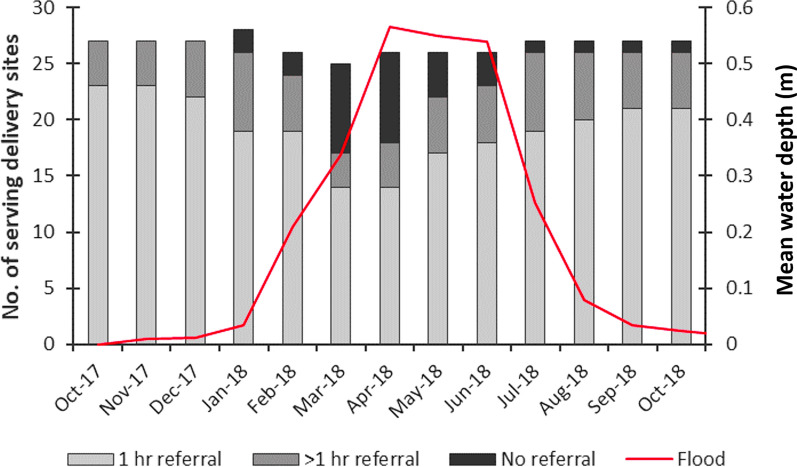
Number of delivery sites being accessed by women each month and their referral capability. The red line indicates modelled floodwater extent as measured across the floodplain study area. A “serving delivery site” refers to a facility which is being accessed by women and has a direct road connection to EmOC so referrals are possible; the number and location of these changes each month as floodwaters cause women to access different facilities

As the floodwave progresses, the referral system is shown to be impacted (Additional file 1: Table S5). The total number of delivery sites able to make referrals and to make timely referrals within an hour decreased with an increase in mean floodwater depth and extent (Figs. 8 and 9). By March 2018, floods had most detrimentally impacted emergency referrals with the proportion of women modelled to have timely referrals dropping to just 23%. There are 25 health facilities operating as the nearest delivery site to women living on the floodplain, but only 17 are able to make a referral to an EmOC site, and only 14 are able to do so within an hour. The mean referral time is only slightly elevated at 35 min, but the maximum referral time increased to 205 min due to floodwater impact.

Delivery sites with the lowest referral times in the dry season were shown to remain consistently accessible with negligible impacts to their referral times throughout the hydrological year, whilst delivery sites with the highest referral times were most impacted. EmOC sites are situated in urban areas, and thus delivery sites located closer to urban areas experienced fewer inundated roads, and had the benefit of denser connectivity of urban roads to mitigate for any closures through the provision of alternate routes. This pattern reflected a disparity in provision of EmOC services to rural and urban populations.

**Fig. 10:**
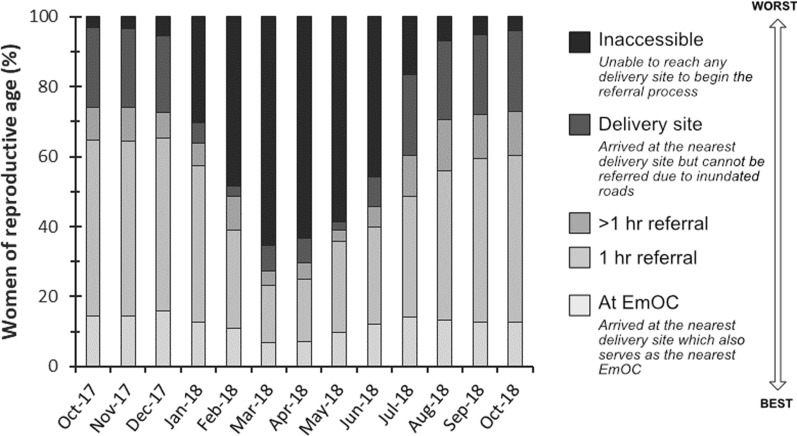
Monthly variation in the percentage of women within the different referral categories

Floods evidently have a detrimental impact on referral coverage and travel times. However, when assessing the relative monthly position of women within the referral system (Fig. 10), it is clear the primary reduction in the proportion of women who could be referred to EmOC in a timely manner occurred due to the complete inability of women to even first reach a delivery site rather than because of an increase in the number of women waiting to be referred by a facility with impacted referral abilities.

## Discussion

### Novelty

This study demonstrated a novel geospatial methodology for incorporating modelled floodwater variables into network-based and raster-based models in order to account for dynamic flood impacts on walking and driving accessibility to maternal health services. To our knowledge, this represents the first time that a hydrodynamic inundation model has been coupled with a raster-based health access model, and the first usage of quantitative hydrodynamic model outputs in network analysis for a low-income country. This work has expanded upon previous geographic access models (e.g., [11, 17, 99, 102]) through the consideration of flood depth and velocity on access to maternal services, developing frameworks that are more physically representative of this important seasonal phenomenon but still remain appropriate for application in data-limited, low-income countries. Most notably, our approach using flood models offers predictive capabilities to health access models that expands the scope to quantify future access, such as under climate change.

The importance of hydrological seasonality in affecting access has been recognised but seasonal methodologies were nearly all limited to creating static dry and wet season maps (e.g., [17]). The explicit incorporation of monthly flood depth and velocity demonstrated that representation of physical hydrological processes is important. Dynamic spatio-temporal variations in floods interact with the location of maternal services and populations to create dynamic spatio-temporal variations in access. The varied temporal onset and resolution of flood impact on different maternal services is one crucial example of an important access phenomenon that would have otherwise been obscured by previous static methodologies. Consequently, the results of this study support the conclusions of Makanga et al. [11] in stating that continuous fluctuations in access resulting from seasonality are unable to be represented by two contrasting scenarios of wet season access and dry season access.

Consideration of floodwater depths and velocities permitted a more detailed conceptual representation of how floodwaters impact driving rather than simply assuming all floodwaters are impassable. Thresholds to define driving accessibility and driving speeds through different depths and velocities were parameterised from safety criteria recommendations and a comprehensive review of vehicular instability literature [90, 92, 100, 101]. Water depth thresholds have been used previously in network analysis [102–105] as it is recognised that the presence of floodwaters does not necessarily preclude passage, especially in emergency situations. In this study, it was deemed inappropriate to assume that women accessing maternal services would walk through floodwaters, so any flooded extent was treated as impassable. However, the raster-based model has a similar capability to utilise depth and velocity data to calculate flood hazard to human stability [92, 106–111] and thus to determine whether flooded areas are safe for passage. This capability is optional because, as demonstrated with this maternal services case study, it may not be appropriate to assume that people can walk through floodwaters where deemed safe. However, it has potential advantages for other scenarios because the incorporation of additional hydrodynamic information better constrains the relative hazard of floodwaters; most notably, this expands the applicability of post-disaster geographical accessibility modelling (e.g., [33]). Ultimately, this framework could become a tool through which local health officers can alter parameters to assess the uncertainty of access projections, to investigate particular health scenarios of interest, and to evaluate proposed health system plans.

Past representation of floods in raster-based models have relied on flood extents derived from optical satellite images [11, 33] due to their free and ready availability. Makanga et al. [11] demonstrated that satellite-derived flood extents could be used to assess access seasonally, as they incorporated daily flood extents from the Dartmouth Flood Observatory alongside precipitation records in their spatio-temporal access model. However, flood extents from optical satellite sensors such as Landsat and MODIS are subject to observational biases that are particularly limiting for application to tropical floodplains [112, 113]. Optical sensors are sensitive to cloud cover which predominates during the wet season; extensive cloud cover above the Barotse Floodplain prevented floodwaters from being visible in MODIS imagery for the entire of March 2018, and others have reported difficulty in obtaining cloud-free imagery for the region [34, 36]. Optical sensors also experience difficulty detecting vegetated waters, as do radar sensors albeit to a lesser extent [114–116]. Most readily-available flood extents rely on algorithms suitable for mapping open waters [115, 117] leading to flood omission where inundated vegetation is present [113]. Satellite-derived flood extents were found to underestimate flood inundation due to emergent vegetation in the central axis of the Barotse Floodplain and in the Luena Valley in previous studies [36, 60, 114]. This detection limitation is problematic as we have identified herein that women’s access to maternal health service is especially sensitive to the persistent vegetated waters in the Luena Valley, thereby emphasising their need to be adequately represented in an access model. Whilst ground-truthing can be used to improve the accuracy of satellite-derived flood extents, the surveys are expensive and time-consuming [36]. Hydrodynamic models thus offer an alternative as they do not suffer from the same observational biases, can provide reliable flood information at the temporal and spatial resolution of the modeller’s choosing, and additionally provide water velocity information for hazard-based approaches. Modelling offers potential new advantages for assessing flood risk on health access, such as the simulation of extreme events, climate change analysis, near-time forecasting, and development of early-warning systems.

### Findings and significance for the Barotse Floodplain

This is the first model detailing geographic access to maternal health services for the Barotse Floodplain, indicating that floods exert a clear control on women’s ability to access crucial maternal health services. The annual floodwave was shown to impact walking times and inter-facility emergency referrals, but at the peak of the floods, the majority of women (65%) were isolated due to floodwater impact and were unable to access any health facility (HF). Rural populations have been shown to utilise services less than their urban counterparts when there are substantial geographic barriers to accessing care [118–120], hence floods are likely to be acting as a significant barrier to healthcare uptake in the Barotse Floodplain, with potential implications of increased maternal and neonatal morbidities and mortalities. In the case of the referral system, our findings show that inabilities to make emergency referrals predominantly arise from floods isolating women and preventing them from reaching a delivery site first (where a referral can take place), rather than impacting referrals between delivery sites and EmOC sites. This suggests that efforts to improve the referral system should first ensure women are able to access facilities, such as through the use of interventions like maternity waiting shelters where at-risk women can stay at an EmOC location ahead of delivery. In order to fully comprehend the local situation, this study’s geographic access data requires interpretation with data on health services utilisation and morbidities and mortalities as well as qualitative data on the lived experiences of women in the region. Such analysis would identify the relative importance of geographic access and flood impacts compared with other variables that affect health access, which would be necessary for any future health intervention planning.

In addition to the maternal services of delivery sites, MWS, and EmOC considered here, the lack of access to any HF for many women during the floodwave also has implications for other mother-baby services, such as uptake of antenatal care. Antenatal care is another effective strategy for reducing maternal mortalities, and a minimum of four visits throughout pregnancy are recommended [121, 122]. Given that flooding occurs between December to August for a total of nine months out of the year, a woman can expect to have her access to maternal services impacted at some point throughout her pregnancy. Strong feedback loops can emerge between the perception of flooding and healthcare-seeking behaviours of women [56, 123], so care needs to be taken to ensure that: (1) women are educated on the necessity of antenatal care and skilled professional deliveries so that they are motivated to seek out these services [121, 124] ; (2) the healthcare quality of facilities is maintained throughout flood months to avoid dissatisfaction and a perception that quality is poor year-round [124]; (3) the impacts of floods on access to maternity services are mitigated, such as through the provision of community health workers who can extend access to those located in the most remote regions [125].

Our spatio-temporal approach identified the communities of women on the Barotse Floodplain who experienced exceptional difficulties or isolation in accessing maternal services during flood months, in addition to the mechanism behind why floods had such a disproportionate impact on their access. It is shown that floods impacted women’s access to EmOC and MWS services in December 2017, a month earlier than floods impacted access to delivery sites. This temporal variation is caused by fewer facilities offering EmOC and MWS, which creates geographic vulnerabilities for specific locations of women in the Luena Flats and on the floodplain near Kalabo. A total of 40 health facilities offer delivery sites, and the placement of delivery sites within the existing health infrastructure is optimal as indicated by the pattern of women’s access to the nearest health facility being identical to the pattern of women’s access to the nearest delivery site (Fig. 6). However, only 22 facilities offer MWS, and only 10 offer EmOC. In the Luena Flats, the lack of a MWS situated on the true-right floodplain escarpment prevents many women from being able to access a MWS for the majority of the year. The Luena Flats floods earlier than the Barotse Floodplain and floodwaters persist for longer, preventing women from crossing the valley to reach a MWS. Near to Kalabo, there are 3,288 women living on the floodplain who are geographically restricted within the confinements of the Zambezi. These women rely on connecting roads through to Kalabo to access any EmOC and MWS, but these roads become inundated in December 2017 due to the earlier flooding of the Luanginga Valley. As shallow floodwaters in the valley persist from August 2018 onwards, these women remain unable to access any EmOC and MWS. The identification of this finding permits further investigation into interventions that could potentially be conducted to resolve this inequity in access.

Confidence in the access model findings were ascertained through comparison with available literature. The flood model well-recreated the typical inundation pattern of the annual floodwave [60, 114] which resulted in the access models reproducing key accessibility patterns that have been previously reported; these included the impassability of roads in the Luanginga Valley from December onwards [126], and the continued presence of floodwaters in August that impede access [127].

### Limitations

This study necessarily simplified the complexity of women’s access to maternal services, and the travel times presented are estimates rather than absolute and precise. The model is explicitly a representation of the physical ability to reach health services, so assumes that people decide to seek care immediately and receive appropriate care at arrival to a facility. This ignores complex delays in seeking care resulting from human decision-making, and delays and problems of receiving good care such as a lack of available staff [27, 56, 121, 123, 128–132].

The available population and health facility data used in this study also presented additional limitations. The usage of HRSL population density data assumed that the Lozi population remained static throughout the year. However, the Lozi people are semi-nomadic and will move between locations depending on flood stage. As no data were available to characterise these movements, it was not possible to account for these yearly migrations. Therefore, there is some uncertainty regarding the location and number of women in different areas of the floodplain in the wet season. The available published health facility data were a decade old and categorised only three maternal services: delivery site, EmOC, and maternity waiting shelter. This introduced some uncertainty about the number and location of maternal services in the study area. After the study’s completion, it was communicated that all rural and urban health centres offer basic emergency obstetric care (BEmOC), whilst hospitals only offer comprehensive emergency obstetric care (CEmOC). Additional classification of facilities into BEmOC and CEmOC offers additional sophisticated insights into emergency maternal care that should be used in subsequent analyses for the region.

The study also made assumptions that add uncertainty to the modelled travel times. In the network-based model, driving speeds are assigned based on government legislation. However, road condition influences real-world driving speeds, with the modelled driving speeds optimistic for roads in poor condition. Road condition is very variable on and surrounding the Barotse Floodplain; however, this information is difficult to ascertain from satellite imagery alone, hence why distinctions in road condition were not made. Additionally, it is assumed that emergency 4WD vehicles are present and immediately able to make emergency referrals. Whilst a common assumption, in reality, these vehicles are often unavailable in many parts of sub-Saharan Africa due to their upkeep and fuelling expenses, and they are often utilised in other tasks [19, 133]. The model also assumes that all facilities have operational communications equipment with which to communicate referrals with [46, 134]. Consequently, women may experience delays in waiting for appropriate transport to refer them to EmOC locations. Similarly, the raster-based model assumes women can walk to their nearest maternal services; however, some women in labour may be incapable of movement [123] and thus reliant and delayed waiting for alternative modes of transport. For women that can walk, the model attempts to simulate impeded walking by parameterising travel speeds to be lower than average. However, a limitation is that these speeds remain static and there is no accounting for potential decreases in walking speeds due to exhaustion after walking long distances. In general, there is a complexity of variables affecting an individual’s walking speeds [135, 136], including factors such as the weather and time of day, but these were not modelled herein. As a result, in both the vector- and raster-based model, these assumptions and resulting limitations mean that the travel times modelled are best-case-scenario, optimal travel times.

Another limitation of the raster-based model was that it assumed women solely walk to maternal services. In the Barotse Floodplain, this unimodular representation of access doesn’t account for journeys often using oxen-carts or dugout canoes for parts of journeys in combination with walking. Insufficient data were available to adequately characterise and conceptualise this real-world transport behaviour into a model. Oxen-carts have similar travel speeds to walking so the differences in travel times and thus uncertainty is assumed to be minimal. However, dugout canoes operationalise areas that would otherwise be completely inaccessible to walking, thus can facilitate access where land-based transport is unavailable. As a consequence, some populations may be modelled as inaccessible when they may otherwise still be able to access maternal services through the use of a canoe. In the Barotse Floodplain, dugout canoes are commonplace and used on canals, rivers, and on open, unvegetated floodwaters. There is a dearth of research incorporating boats as a mode of transport in access models [42–45] which prevented their incorporation into the raster-based access model. This study has emphasised the capability of health access models to simulate boats as a focus for ongoing and future research.

## Conclusions

Annual floods are common in low-income countries with seasonal climates, but assessing the impact of these floods on women’s access to maternal health services has been hampered by the lack of models able to represent the spatio-temporal impacts of floods on access. The necessity of geographic health access models able to represent this important spatio-temporal barrier is becoming increasingly more important under climate change. This study created novel geospatial models that were able to account for the impacts of floods on women’s walking access to healthcare facilities, and driving times for inter-facility referrals. Floods were shown to evidently have a strong control over women’s access to maternal services, with access asynchronously varying depending on flood stage, the location of women, and the positions of maternal services. Modelling access in this way has positive implications for addressing existing health inequities by providing evidence to facilitate interventions designed to increase all-weather year-round access to maternal health services, and to reduce the disproportionate impacts of floods on the most vulnerable communities of women.

## Supplementary Information

**Additional file 1: Table S1. **Reclassification of OpenStreetMap data. **Table S2.** Vehicular instability thresholds as recommended by the Australian Rainfall and Runoff Project [131]. **Figure S1.** Boxplot showing the variability in the monthly sum of precipitation as recorded between 1993-2010 for the Upper Zambezi catchment. **Table S3.** Monthly average depths, velocities, and waterbody and flood extent across the study area. **Table S4. **The number and percentage of women (total *n *= 8,130) who are inaccessible and who have timely walking access to maternal services each month. **Table S5. **The number and percentage of women (total *n *= 8,130) who are unable to be referred by road to EmOC and those who have timely vehicular referral access to EmOC each month. **Table S6. **Description and source of all data used in this study.

## Data Availability

Additional file 1: Table S6 provides a description and source of all data used in this study. The waterbodies shapefile and monthly rasters of floodwater depth and velocity are openly available from the University of Leeds Data Repository: 10.5518/1362. The roads dataset as used in this study (consisting of OpenStreetMap data fused with manually delineated data) are also openly available from the University of Leeds Data Repository: 10.5518/1362. OpenStreetMap is licensed under the Open Data Commons Open Database License (https://www.openstreetmap.org/copyright) and is freely-accessible to use. The HRSL population data for women of reproductive age are freely available from the Human Data Exchange: https://data.humdata.org/organization/facebook?q=high%20resolution%20population%20density&ext_page_size=100. Geolocation information on health facilities in Zambia are freely available from the Ministry of Health Master Facility List: https://mfl.moh.gov.zm/facility/index. Details of the services available in Zambian health facilities as of 2012 are available from the 2012 List of Health Facilities in Zambia report, as cited.

## Abbreviations

GIS: Geographical information systems
HF: Healthcare facility
EmOC: Emergency obstetric care
BEmOC: Basic emergency obstetric care
CEmOC: Comprehensive emergency obstetric care
ITCZ: Inter-Tropical Convergence Zone
HRSL: High Resolution Settlement Layer
4WD: Four-wheel drive vehicles
MFL: Zambia’s Master Facility List
OSM: OpenStreetMap
GPS: Global positioning system
DEM: Digital elevation model
WARMA: Water Resources Management Agency
MWS: Maternity waiting shelter
D: Floodwater depth
V: Floodwater velocity
