# Supplementary material for: Impacts of seasonal flooding on geographical access to maternal healthcare in the Barotse Floodplain, Zambia
Source: Int J Health Geogr. 2023 Jul 31;22:17. doi: 10.1186/s12942-023-00338-3 (PMC10391775; doi:10.1186/s12942-023-00338-3)
Supplement: Supplementary file 1 — Additional file 1: Table S1. Reclassification of OpenStreetMap data. Table S2. Vehicular instability thresholds as recommended by the Australian Rainfall and Runoff Project [131]. Figure S1. Boxplot showing the variability in the monthly sum of precipitation as recorded between 1993-2010 for the Upper Zambezi catchment. Table S3. Monthly average depths, velocities, and waterbody and flood extent across the study area. Table S4. The number and percentage of women (total n = 8,130) who are inaccessible and who have timely walking access to maternal services each month. Table S5. The number and percentage of women (total n = 8,130) who are unable to be referred by road to EmOC and those who have timely vehicular referral access to EmOC each month. Table S6. Description and source of all data used in this study. [file 12942_2023_338_MOESM1_ESM.docx]

**Additional file**

**Additional file Table 1.** Reclassification of OpenStreetMap data.

| **OpenStreetMap classification** | **Recode** |
| --- | --- |
| Trunk | Primary road |
| Primary | Secondary road |
| Secondary | Secondary road |
| Tertiary | Secondary road |
| Track | Track |
| Residential | Track |
| Unclassified | Track |
| Service | Track |
| Path | Track |
| Footway | Removed in network model (track in walking model only) |
| Steps | Removed in network model (track in walking model only) |

**Additional file Table 2:** Vehicular instability thresholds as recommended by the Australian Rainfall and Runoff Project (131).

| **Vehicle type** | **Maximum flood depth stability criteria (m)** | **Maximum flood velocity stability criteria (m s^-1^)** | **Maximum combined flood depth and velocity stability criteria (m s^-1^)** |
| --- | --- | --- | --- |
| Small passenger vehicle | 0.3 | 3.0 | 0.3 |
| Large passenger vehicle | 0.4 | 3.0 | 0.45 |
| Four wheeled drive (4WD) | 0.5 | 3.0 | 0.6 |


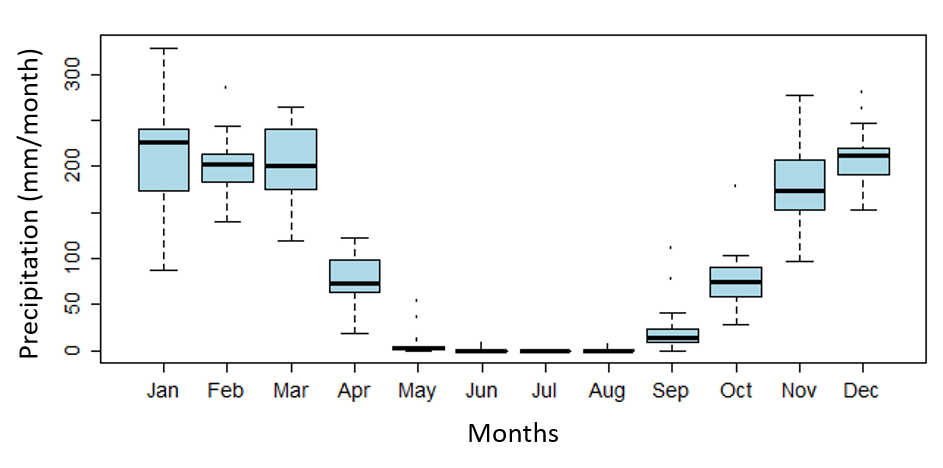


**Additional file Figure 1.** Boxplot showing the variability in the monthly sum of precipitation as recorded between 1993-2010 for the Upper Zambezi catchment.

**Additional file Table 3.** Monthly average depths, velocities, and waterbody and flood extent across the study area.

| **Month** | **Mean depth (m)** | **Mean velocity (m s^-1^)** | **Waterbody and flood extent (km^2^)** |
| --- | --- | --- | --- |
| ***October 2017*** | 0.0098 | 0.0002 | 31.02 |
| ***November 2017*** | 0.0112 | 0.001 | 190.51 |
| ***December 2017*** | 0.0345 | 0.0025 | 431.04 |
| ***January 2018*** | 0.21 | 0.0189 | 1751.57 |
| ***February 2018*** | 0.3377 | 0.0392 | 2516.96 |
| ***March 2018*** | 0.5659 | 0.0699 | 3511.52 |
| ***April 2018*** | 0.5497 | 0.0659 | 3336.54 |
| ***May 2018*** | 0.5388 | 0.0641 | 3206.53 |
| ***June 2018*** | 0.2522 | 0.0196 | 2388.34 |
| ***July 2018*** | 0.0795 | 0.0025 | 1230.08 |
| ***August 2018*** | 0.0352 | 0.001 | 577.25 |
| ***September 2018*** | 0.0238 | 0.0009 | 372.16 |
| ***October 2018*** | 0.0161 | 0.0008 | 274.25 |

**Additional file Table 4.** The number and percentage of women (total *n* = 8,130) who are inaccessible and who have timely walking access to maternal services each month.

| ***Month*** | ***Inaccessibility*** | | | | | | ***Timely access (< 2 hrs)*** | | | | | |  |
| --- | --- | --- | --- | --- | --- | --- | --- | --- | --- | --- | --- | --- | --- |
|  | **Delivery** | | **EmOC** | | **MWS** | | **Delivery** | | **EmOC** | | **MWS** | |  |
|  | **%** | **n** | **%** | **n** | **%** | **n** | **%** | **n** | **%** | **n** | **%** | **n** |  |
| ***October 2017*** | 3 | 256 | 3 | 256 | 3 | 256 | 55 | 4,445 | 19 | 1,507 | 24 | 1,971 |  |
| ***November 2017*** | 3 | 269 | 3 | 269 | 3 | 269 | 55 | 4,444 | 19 | 1,507 | 24 | 1,970 |  |
| ***December 2017*** | 5 | 438 | 45 | 3,654 | 51 | 4,146 | 51 | 4,121 | 17 | 1,396 | 22 | 1,788 |  |
| ***January 2018*** | 30 | 2,456 | 59 | 4,798 | 65 | 5,285 | 42 | 3,394 | 14 | 1,149 | 18 | 1,426 |  |
| ***February 2018*** | 48 | 3,929 | 63 | 5,156 | 68 | 5,527 | 36 | 2,963 | 14 | 1,140 | 17 | 1,378 |  |
| ***March 2018*** | 65 | 5,313 | 76 | 6,200 | 74 | 6,039 | 29 | 2,362 | 14 | 1,104 | 16 | 1,337 |  |
| ***April 2018*** | 63 | 5,141 | 71 | 5,787 | 72 | 5,871 | 29 | 2,363 | 14 | 1,125 | 16 | 1,339 |  |
| ***May 2018*** | 59 | 4,764 | 67 | 5,426 | 71 | 5,736 | 29 | 2,397 | 14 | 1,125 | 17 | 1,346 |  |
| ***June 2018*** | 46 | 3,728 | 60 | 4,869 | 66 | 5,327 | 37 | 2,975 | 14 | 1,148 | 17 | 1,365 |  |
| ***July 2018*** | 16 | 1,340 | 51 | 4,132 | 57 | 4,621 | 46 | 3,745 | 15 | 1,239 | 20 | 1,601 |  |
| ***August 2018*** | 7 | 562 | 45 | 3,681 | 45 | 3,681 | 51 | 4,114 | 17 | 1,356 | 21 | 1,736 |  |
| ***September 2018*** | 5 | 415 | 45 | 3,627 | 45 | 3,627 | 53 | 4,304 | 17 | 1,381 | 22 | 1,817 |  |
| ***October 2018*** | 4 | 330 | 44 | 3,600 | 44 | 3,600 | 53 | 4,328 | 17 | 1,396 | 23 | 1,838 |  |

**Additional file Table 5.** The number and percentage of women (total *n* = 8,130) who are unable to be referred by road to EmOC and those who have timely vehicular referral access to EmOC each month.

| ***Month*** | ***Inaccessibility (no referrals)*** | | ***Timely access (referrals within 1 hr)*** | |
| --- | --- | --- | --- | --- |
|  | **%** | **n** | **%** | **n** |
| ***October 2017*** | 26 | 2,098 | 65 | 5,251 |
| ***November 2017*** | 26 | 2,112 | 64 | 5,244 |
| ***December 2017*** | 27 | 2,227 | 65 | 5,309 |
| ***January 2018*** | 36 | 2,937 | 57 | 4,666 |
| ***February 2018*** | 51 | 4,176 | 39 | 3,163 |
| ***March 2018*** | 73 | 5,904 | 23 | 1,892 |
| ***April 2018*** | 70 | 5,721 | 25 | 2,024 |
| ***May 2018*** | 61 | 4,961 | 36 | 2,907 |
| ***June 2018*** | 54 | 4,415 | 40 | 3,245 |
| ***July 2018*** | 40 | 3,232 | 49 | 3,949 |
| ***August 2018*** | 29 | 2,381 | 56 | 4,546 |
| ***September 2018*** | 28 | 2,284 | 59 | 4,833 |
| ***October 2018*** | 27 | 2,213 | 60 | 4,905 |

**Additional file Table 6.** Description and source of all data used in this study.

| **Data layer** | **Description** | **Publication date** |  | **Data type and resolution** | **Source** |
| --- | --- | --- | --- | --- | --- |
| ***Roads*** | Roads manually delineated through visual inspection of Google Earth and Microsoft Bing Maps satellite image, fused with OpenStreetMap data. *See Supplementary Table 1 for details of the classification.* | July 2021 |  | Vector polyline | Fused roads dataset as used in this study available from the University of Leeds: [https://doi.org/10.5518/1362](https://eur03.safelinks.protection.outlook.com/?url=https%3A%2F%2Fdoi.org%2F10.5518%2F1362&data=05%7C01%7Cgy16e3m%40leeds.ac.uk%7Cc6739bbc04cb4ceb67ce08db65b541f5%7Cbdeaeda8c81d45ce863e5232a535b7cb%7C1%7C0%7C638215601434227335%7CUnknown%7CTWFpbGZsb3d8eyJWIjoiMC4wLjAwMDAiLCJQIjoiV2luMzIiLCJBTiI6Ik1haWwiLCJXVCI6Mn0%3D%7C3000%7C%7C%7C&sdata=Vyam3ew7LVxGUBExqLjdoiITXZMRP4u52Me%2BjU%2F8eAc%3D&reserved=0)  *OpenStreetMap* *contributors* for the OpenStreetMap data, available from: <https://www.openstreetmap.org/> |
| ***Public health facilities*** | Geolocated points detailing the locations of public health facilities, supplemented with information on maternal services where available from the 2012 List of Health Facilities in Zambia. Missing information was assumed by applying common services typically offered by the same facility type. | August 2021 |  | Vector points | Zambia’s Ministry of Health Master Facility List: <https://mfl.moh.gov.zm/>  2012 List of Health Facilities in Zambia: <https://www.moh.gov.zm/docs/facilities.pdf> |
| ***Women of reproductive age*** | Gridded population density data of women of reproductive age from the High Resolution Settlement Layer. Additional details available from: <https://docs.digitalearthafrica.org/en/latest/sandbox/notebooks/Datasets/High_Resolution_Population_Density.html> | August 2021 |  | Raster, 30 m spatial resolution | Facebook Connectivity Lab and CIESIN: <https://www.ciesin.columbia.edu/data/hrsl/> |
| ***Permanent waterbodies*** | Geolocated polylines of the positions of waterbodies, with average widths of each waterbody provided in the attribute table. | November 2022 |  | Vector polyline | Willis et al., 2022: <https://doi.org/10.1029/2021WR030107> |
| ***Floodwater depth and velocity*** | LISFLOOD-FP hydrodynamic inundation model (version 8.1) set-up for the Barotse Floodplain in a separate study conducted by co-authors of this paper. Data were taken for each month between October 2017 and October 2018. This study did not create the flood model and instead directly used its outputs without modification. *See Willis et al., 2022 for details on the inputs and parameters of the model.* | November 2022 |  | Raster, 100m spatial resolution, monthly temporal resolution | Willis et al., 2022: <https://doi.org/10.1029/2021WR030107> |
